# Efficacy and Safety of Ferric Chloride in Controlling Hepatic Bleeding; An Animal Model Study

**DOI:** 10.5812/hepatmon.18652

**Published:** 2014-06-09

**Authors:** Saeed Nouri, Mohammad Reza Sharif

**Affiliations:** 1 Chemical Injuries Research Center, Baqiyatallah University of Medical Sciences, Tehran, IR Iran; 2 Trauma Research Center, Kashan University of Medical Sciences, Kashan, IR Iran

**Keywords:** Hemostasis, Ferric Chloride, Liver

## Abstract

**Background:**

Controlling parenchymal hemorrhage especially in liver parenchyma, despite all the progress in surgical science, is still one of the challenges surgeons face saving patients’ lives and there is a research challenge among researchers in this field to introduce a more effective method.

**Objectives:**

This study attempts to determine the haemostatic effect of ferric chloride and compare it with that of the standard method (suturing technique) in controlling bleeding from liver parenchymal tissue.

**Materials and Methods:**

In this animal model study 60 male Wistar rats were used. An incision, two centimeters (cm) long and half a cm deep, was made on each rat’s liver and the hemostasis time was measured once using ferric chloride with different concentrations (5%, 10%, 15%, 25% and 50%) and then using the control method (i.e. controlling bleeding by suturing). The liver tissue was examined for pathological changes.

**Results:**

The hemostasis time of ferric chloride concentration groups was significantly less than that of the control group (P value < 0.001). The pathologic examination showed the highest frequency of low grade inflammation based on the defined pathological grading.

**Conclusions:**

Ferric chloride is an effective haemostatic agent in controlling liver parenchymal tissue hemorrhage in an animal model.

## 1. Background

The control of a solid organ’s hemorrhage like liver, due to its rich vascular network, is a challenging task, even in the operating room. The main problem in the hemostasis of liver is the sinosoidal structures of this organ, where blood vessels are too small to be closed by using routine surgery techniques (1-4). On the other hand, the number of operations in which liver needs to be cut, such as metastatectomy and liver trauma, is increasing day by day (5). The high morbidity and mortality rates of liver injuries are attributed to too large volume of blood lost and extensive control of bleeding imposed to the patient (6). This has motivated many studies and led to the introduction of new techniques like intermittent clamping of the portal triad (7) for controlling liver bleeding, and the goal of these studies is to introduce a treatment method for liver bleeding control, that can prevent the complete resection of the bleeding part of the liver (8-11). Ferric chloride is a dark brown chemical agent with formula FeCl_3_ and acidic property. Ferric chloride is widely used as a protein coagulant in purifying water (12). This property thereof, with regard to the significant amount of proteins in blood, makes ferric chloride a very strong haemostatic agent. Ferric chloride exerts its haemostatic effect through a chemical reaction with blood, and this property makes ferric chloride a very efficient haemostatic agent, without the need for normal body haemostatic system to exert its effect and even in the patients with abnormal body haemostatic system, it adequately satisfies surgeons’ need to control bleeding (13-15). Although the haemostatic effect of this agent has already been found (16), evaluating this effect of ferric chloride in controlling liver bleeding and comparing it with the standard method (suturing technique) was not performed before.

## 2. Objectives

This study attempts to determine the haemostatic effect of ferric chloride and compare it with that of the standard method (suturing technique) in controlling bleeding from liver parenchymal tissue.

## 3. Materials and Methods

### 3.1. Study Setting and Animals

This study took place at Kashan University of Medical Sciences from August 2013 to December 2013. In this experimental study 60 male Wistar rats, weighing 180-230 grams, were randomly divided into 6 groups, 10 rats in each group. All animals received care in compliance with the American Convention on Animal Care and the study was approved by the Institutional Ethics Committee at Kashan University of Medical Sciences. One week before the study, all the rats were kept and fed in similar situations.

### 3.2. Surgery

**Figure 1.**
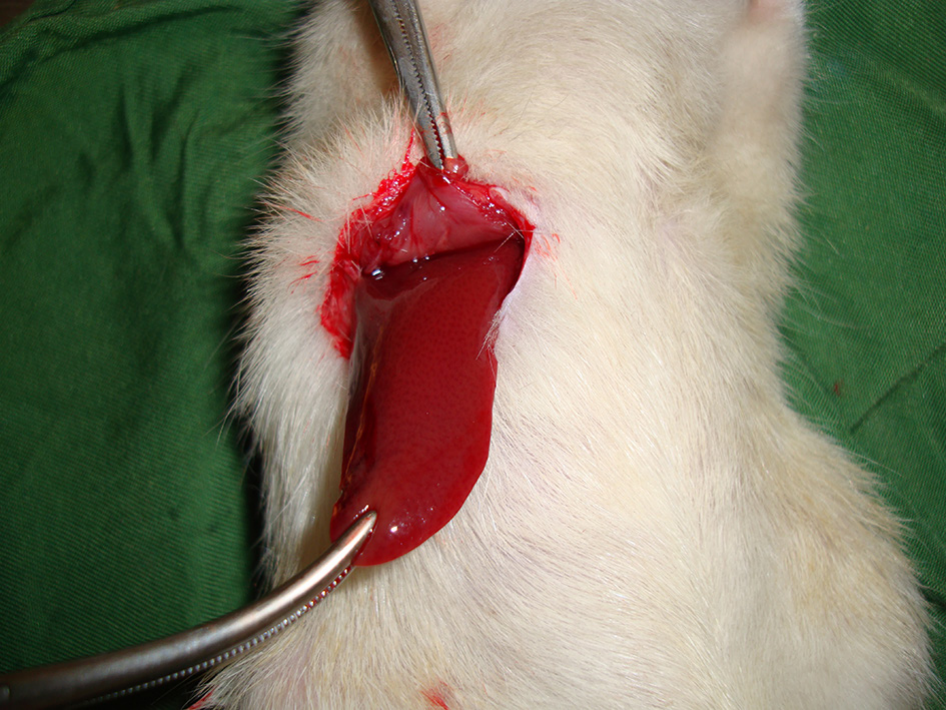
Extracting the Liver Lobe from the Abdominal Cavity

The rats were anesthetized by kethamine (10 mg/kg). Then the qutaneous and subqutaneous layers in the abdominal zone were opened, and after determining the anatomical position of the liver, the liver lobe was extracted from the abdominal cavity (Figure 1). Next, a two-centimeter long and half-a-centimeter deep cut was made on the liver by a scalpel (the depth was determined by a mark made on the scalpel half a cm from the tip, and the length was measured with a ruler on the liver).

### 3.3. Ferric Chloride Administration and Haemostatic Time Measurement

**Figure 2.**
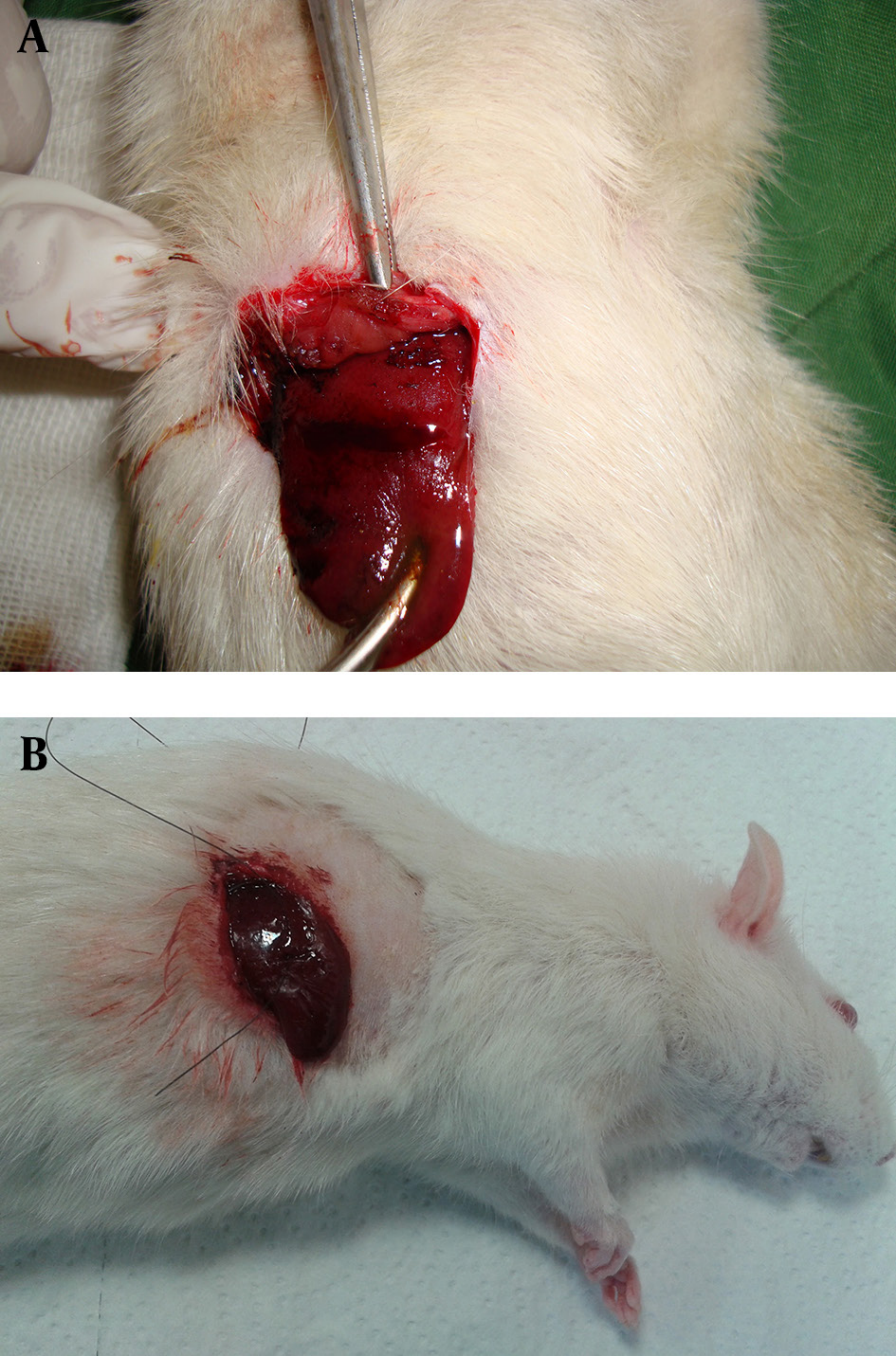
Control of the Liver Bleeding by (A1) Ferric Chloride 50% and (A2) Suturing Technique

The ferric chloride was purchased from Merck, (Darmstadt, Germany 103814) and diluted with distilled water. After that, 50%, 25%, 15%, 10% and 5% concentrations of ferric chloride were applied onto the incision in the rats’ livers by a swap. Similar swaps were used to remove the same amount of solution each time and each swap entered the solution only once and was applied on the incision only once. In fact, each concentration of ferric chloride was used in one of the groups, and the times of hemostasis were measured by chronometer (Figure 2). In this study, the haemostatic time was considered the time required for the complete stopping of the bleeding with no blood discharge from the incision site. The mean of the ten measured times was considered as the haemostatic time for each concentration. Suturing (the standard method), was used in the control group to be compared with the results of the ferric chloride concentrations. The time of liver hemostasis was measured using sutures (all the stitches were made by one surgeon) on the livers of the ten rats, that were kept and fed in similar situations with the other groups, and the mean of the ten obtained times was compared with the results of different concentrations of ferric chloride. After controlling liver hemorrhage, subcutaneous and skin were closed again, and to prevent infection, each rat received 50 mg of keflin through an intraperitoneal injection.

### 3.4. Pathological Study

**Figure 3.**
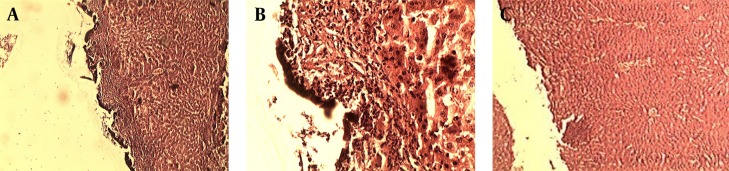
Pathological Effect of Ferric Chloride and Suturing Technique on the Liver Tissue

After one week the rats were anesthetized by kethamine and were placed in a supine position on the operating table. Then an incision was made on the previous site and the rats’ livers were resected, immediately placed and fixed in formalin, and sent to the laboratory for the pathology report (Figure 3). Studying the pathological effect of ferric chloride on the liver tissue was performed through staining with haematoxylin and eosin (H and E) by light microscopy. Based on the defined pathological grading, pathology results were classified into 6 grades: 0. No change; 1. Minor inflammatory infiltration without edema; 2. Mild to moderate inflammatory infiltration with mild edema; 3. Mild to moderate inflammatory infiltration with moderate edema; 4. Moderate inflammation with neutrophils scattered and diffuse edema; 5. Severe inflammation of the tissue and edematous changes, fibrosis and hemorrhage.

Studying the pathological effect of ferric chloride and Suturing Technique on the liver tissue was performed through staining with haematoxylin and eosin (H and E) by light microscopy. (A1) Acidic property of ferric chloride after reaction with blood proteins creates a barrier by coagulated proteins, and prevents the outflow of blood from vessels. On the other hand, it prevents the ferric chloride from entering the vessels, and as a result, it does not allow potential systemic complications of ferric chloride to appear. (A2) Higher magnification (Pathological Grade 2 Caused by Ferric Chloride 50%). (B1) Pathological Effect of Suturing Technique on the Liver Tissue. (B2) Higher magnification (Pathological Grade 1 Caused by Suturing Technique).

### 3.5. Statistical Analysis

The obtained data was entered into SPSS software, version 16, and because of the abnormal distribution of variables in Kolmogorov-Smirnov test, the data was analyzed using Kruskal-Wallis test, Mann-Whitney, and also Wilcoxon Signed Ranks test.

## 4. Results

### 4.1. Haemostatic Results

The haemostatic time of the 6 groups are shown in Table 1. In all the groups complete hemostasis occurred, and there was a statistically significant difference between every two haemostatic times (P value < 0.001). The haemostatic times of different concentrations of ferric chloride were significantly less than that of the control group (P value < 0.001).

**Table 1.** The Haemostatic Time After Using Different Concentrations of Ferric Chloride and Suturing Technique in The Liver Parenchyma ^a^

	Ferric Chloride 5%, time	Ferric Chloride 10%, time	Ferric Chloride 15%, time	Ferric Chloride 25%, time	Ferric Chloride 50%, time	Suture, time	P value
**Groups, Mean **±** SD**	40.60 ± 7.66	30.70 ± 4.11	22.30 ± 4.94	13.70 ± 3.16	7.60 ± 2.22	91.30 ± 7.28	< 0.001

^a^ Date are presented as Mean ± SD.

### 4.2. Pathological Results

At concentrations of 5%, 10% and 15% ferric chloride groups and suture group, grade one was the most common pathological grade. Also, in the 25% and 50% ferric chloride groups, pathological grade two was the most common grade. Grade zero, three, four and five were not seen in any of the study groups (Table 2).

**Table 2.** The Frequency of Liver Pathological Grade (Grades Zero to Five Based on the Severity of Pathological Inflammation) Seven Days after Exposure to Different Concentrations of Ferric Chloride and Suturing Technique

Pathological Grade	Ferric Chloride 5%	Ferric Chloride 10%	Ferric Chloride 15%	Ferric Chloride 25%	Ferric Chloride 50%	Suture
**Grade 1, No. (%)**	10 (100)	10 (100)	10 (100)	3 (30)	2 (20)	10 (100)
**Grade 2, No. (%)**	0 (0)	0 (0)	0 (0)	7 (70)	8 (80)	0 (0)
**Total, No. (%)**	10 (100)	10 (100)	10 (100)	10 (100)	10 (100)	10 (100)

## 5. Discussion

In this study the obtained data showed that ferric chloride, compared with the standard method used in the control method (with a deep suture of the liver parenchymal) needs significantly less time to exert its haemostatic effect. Currently in treatment centers, choice techniques used to minimize bleeding during liver surgery are based on personal preference, physicians’ experience and facilities available. The standard method used to control bleeding from liver lacerations clamps the vascular area by deep stitches or the pack method (17-21). It should be considered that liver bleeding control with sutures can cause more injuries, both parenchymal and ischemic, in the normal liver tissues. On the other hand, the liver parenchymal tissue is not a suitable tissue for stitching, and with a low-experienced surgeon, the sutures will exacerbate the rupture of the liver parenchyma. Pack method also has the risk of re-bleeding and abdominal compartment syndrome, which will impose additional surgery to the patient. Intermittent clamping of the portal triad is also associated with more bleeding than continuous clamping (7). Local agents, like fibrin sealants, provide a matrix for endogenous coagulation, and stimulate hemostasis on the cut surface of the liver parenchymal. In fact, to exert their functions, they require normal homeostatic systems, which is a big disadvantage of this drug class, because many of the issues that require surgery, such as cirrhosis of the liver due to liver dysfunction, impairs the homeostatic function of the body (22). In a large, randomized, controlled trial in 300 patients undergoing partial liver resection, Figueras and his colleagues found no difference in the total blood loss, transfusion requirements, or postoperative morbidity between patients treated with fibrin sealants (n = 150) and a control group without fibrin sealants (n = 150) (23). Aprotinin and tranexamic acid were shown to result in a significant reduction in blood loss and transfusion requirements of around 30% to 40%. Due to the recent safety concerns, especially a high risk of renal failure and perioperative death in patients given aprotinin during cardiac surgery, marketing of aprotinin has recently been suspended (24).

Few studies have been conducted on local haemostatic agents to indicate the usefulness of these materials in reducing haemostatic time, and patients’ need for blood and blood products transfusion, leading to improved prognosis of patients after a liver surgery (25-29). Ferric chloride, unlike well-known haemostatic agents, exerts its haemostatic effect through a chemical reaction with blood, and this property makes ferric chloride a very efficient haemostatic agent, which does not need normal body haemostatic system to exert its effect (30, 31). Besides, the acidic property of ferric chloride should be considered too; this chemical agent, reacting with blood proteins, can create a barrier by coagulated proteins preventing the outflow of blood from vessels. On the other hand, it prevents the ferric chloride from entering the vessels, which will not allow potential systemic complications of ferric chloride to appear, like ferric chloride-induced thrombosis in liver capillaries (Figure 3) (30, 31). In order to examine the potential complications of ferric chloride asserted by previous researchers, in this study, after the liver tissues were exposed to ferric chloride, they were sent to the laboratory for pathological examination, and the pathological effect of this haemostatic agent was evaluated. In order to determine the inflammation of the liver, caused by exposure to the ferric chloride as a foreign body, a pathological grading was used. This grading was scored from zero to five, according to the severity of the inflammation, by an experienced pathologist. Pathological reports showed that the ferric chloride, even at very high concentration (50%) did not cause any inflammation greater than grade two, and the immune system reaction to this haemostatic agent does not differ much from the standard method (sutures) used in controlling superficial bleeding. In another study, Nouri et al. sought the haemostatic effect of ferric sulphate (which acts like ferric chloride) on external bleeding. They have reported that ferric sulphate is an effective haemostatic agent, and noted that skin tissue of Wistar rats had a slight inflammatory reaction to ferric sulphate as a foreign body (31). Kim and Rethnam stated that a good haemostatic material is the one that stops bleeding in the shortest possible time, one that is easily portable and compatible with life, imposes minimum complication to the patient, does not interfere with tissue healing, with a reasonable price (32). Considering the definition of a haemostatic agent provided by these researchers, the unique features of ferric chloride mentioned, such as not requiring normal haemostatic system for function unlike other haemostatic agents, make this chemical substance an extremely effective topical haemostatic agent for controlling liver parenchymal tissue bleeding, along with other methods.
